# Specific Humoral Immunity versus Polyclonal B Cell Activation in *Trypanosoma cruzi* Infection of Susceptible and Resistant Mice

**DOI:** 10.1371/journal.pntd.0000733

**Published:** 2010-07-06

**Authors:** Marianne A. Bryan, Siobhan E. Guyach, Karen A. Norris

**Affiliations:** Department of Immunology, School of Medicine, University of Pittsburgh, Pittsburgh, Pennsylvania, United States of America; Institute of Tropical Medicine (NEKKEN), Japan

## Abstract

**Background:**

The etiologic agent of Chagas Disease is *Trypanosoma cruzi*. Acute infection results in patent parasitemia and polyclonal lymphocyte activation. Polyclonal B cell activation associated with hypergammaglobulinemia and delayed specific humoral immunity has been reported during *T. cruzi* infection in experimental mouse models. Based on preliminary data from our laboratory we hypothesized that variances in susceptibility to *T. cruzi* infections in murine strains is related to differences in the ability to mount parasite-specific humoral responses rather than polyclonal B cell activation during acute infection.

**Methodology/Principal Findings:**

Relatively susceptible Balb/c and resistant C57Bl/6 mice were inoculated with doses of parasite that led to similar timing and magnitude of initial parasitemia. Longitudinal analysis of parasite-specific and total circulating antibody levels during acute infection demonstrated that C57Bl/6 mice developed parasite-specific antibody responses by 2 weeks post-infection with little evidence of polyclonal B cell activation. The humoral response in C57Bl/6 mice was associated with differential activation of B cells and expansion of splenic CD21^high^CD23^low^ Marginal Zone (MZ) like B cells that coincided with parasite-specific antibody secreting cell (ASC) development in the spleen. In contrast, susceptible Balb/c mice demonstrated early activation of B cells and early expansion of MZ B cells that preceded high levels of ASC without apparent parasite-specific ASC formation. Cytokine analysis demonstrated that the specific humoral response in the resistant C57Bl/6 mice was associated with early T-cell helper type 1 (Th1) cytokine response, whereas polyclonal B cell activation in the susceptible Balb/c mice was associated with sustained Th2 responses and delayed Th1 cytokine production. The effect of Th cell bias was further demonstrated by differential total and parasite-specific antibody isotype responses in susceptible versus resistant mice. T cell activation and expansion were associated with parasite-specific humoral responses in the resistant C57Bl/6 mice.

**Conclusions/Significance:**

The results of this study indicate that resistant C57Bl/6 mice had improved parasite-specific humoral responses that were associated with decreased polyclonal B cell activation. In general, Th2 cytokine responses are associated with improved antibody response. But in the context of parasite infection, this study shows that Th2 cytokine responses were associated with amplified polyclonal B cell activation and diminished specific humoral immunity. These results demonstrate that polyclonal B cell activation during acute experimental Chagas disease is not a generalized response and suggest that the nature of humoral immunity during *T. cruzi* infection contributes to host susceptibility.

## Author Summary

Chagas disease, caused by the protozoan parasite *Trypanosoma cruzi*, affects 10–12 million people in Latin America. Patent parasitemia develops during acute disease. During this phase, polyclonal B cell activation has been reported to generate high levels of serum antibody with low parasite specificity, and delayed protective humoral immunity, which is necessary to prevent the host from succumbing to infection. In this manuscript, data show that relatively resistant mice have improved parasite-specific humoral immunity and decreased polyclonal B cell activation compared to susceptible mice. Parasite-specific humoral immunity was associated with differential expansion of B cell subsets and T cells in the spleen, as well as with increased Th1 and decreased Th2 cytokine production. These data suggest that host susceptibility/genetic biases impact the development of humoral responses to infection. Th2 cytokines are generally associated with improved antibody responses. In the context of *T. cruzi* infection of susceptible mice, Th2 cytokines were associated with increased total antibody production concomitant with delayed pathogen-specific humoral immunity. This study highlights the need to consider the effect of host biases when investigating humoral immunity to any pathogen that has reported polyclonal B cell activation during infection.

## Introduction

The protozoan parasite, *Trypanosoma cruzi* is the etiologic agent of Chagas' disease. Chagas disease is a chronic and debilitating syndrome that affects millions of people in Latin America. Infection with *T. cruzi* leads to patent parasitemia and systemic spread of the parasite throughout the host during acute phase disease. Immune control resolves patent parasitemia, but tissue infection persists for the life of the host and leads to chronic phase disease in as many as 30 percent of infected individuals [1]. Due to the difficulties of human studies, the majority of research regarding immune control of parasite infection has been done in experimental murine models, which develop detectable parasitemia during acute infection followed by chronic tissue parasitism that mimics human disease.

Control of *T. cruzi* infection depends on clearance of blood stream parasite through both innate and acquired immune mechanisms. Macrophages, NK cells, T and B lymphocytes, and the production of cytokines, which play key roles in regulating both parasite replication and immune response [2], are required to control parasitemia. The depletion or absence of any given innate or adaptive effector mechanism leads to increased parasitemia and susceptibility to disease [3], [4], [5], [6], [7], [8], [9].

Humoral immunity is important for control of parasite infection as B cell depletion leads to increased parasitemia and mice succumb to otherwise non-lethal infection [7]. Adoptive transfer of antibodies from late stage *T. cruzi* infected mice to naïve mice leads to rapid clearance of parasite from circulation [10]. Transfers of splenocytes from mice that have recovered from acute phase infection to naïve mice confers protection against lethal *T. cruzi* infection, which is abolished by removal of B lymphocytes, but relatively insensitive to T cell or macrophage depletion [11]. Yet, evidence indicates that the majority of B cells are not parasite-specific during early *T. cruzi* infection [12].

Polyclonal B cell activation that leads to hypergammaglobulinemia and delayed specific humoral immune response is generally accepted as a characteristic of acute phase Chagas disease in humans and is reported in rodent experimental models of *T. cruzi* infection [13], [14], [15], [16], [17]. The acute phase polyclonal response to *T. cruzi* infection is associated with delayed specific responses [14]. Different IgG isotypes have been implicated in polyclonal B cell activation and parasite-specific antibody responses [18], [19], [20], [21]. Several parasite encoded mitogenic proteins have been identified, but the role of each has yet to be elucidated [22], [23], [24], [25], [26], [27]. B cell expansion in the spleen and lymph nodes during acute *T. cruzi* infection is associated with polyclonal, rather than specific responses [28], [29]. Fas/FasL mediated apoptosis of parasite specific B cells and immature B cells in the bone marrow (BM) has also been reported in Balb/c mice [30], [31],[32]. Studies in Balb/c XID mice show that the depletion of B cell subsets in this model led to an increased resistance to disease that was associated with improved IFN-γ responses, decreased hypergammaglobulinemia, and a skewed natural antibody repertoire [33], [34], [35]. Limited studies of B cell dynamics during *T. cruzi* infection in resistant versus susceptible mice have been reported [20], [28].

Marginal zone (MZ) and follicular (FO) B cells constitute two functionally and anatomically distinct B cell subsets within the spleen [36], [37], [38], [39], [40]. MZ B cells are located at the marginal sinus of the spleen, making these cells first line responders to pathogens in the blood. MZ B cells are more responsive to TI-antigens, respond quickly with natural antibody responses, and generate short-term plasma cells [41]. MZ are also able to migrate to the follicles and participate in germinal center T cell dependent (TD) reactions [42]. FO B cells circulate through the lymph and are found in B cell follicles of the spleen. FO B cells respond to TD antigen and can become long-term plasma cells or memory B cells [43]. Due to their differential location and function, these two B cell subsets can have distinct roles in the development of specific versus polyclonal B cell responses to pathogens [36], [37], [44], [45].

While mouse models have been informative for analysis of immune responses to *T. cruzi* infection, inbred mouse strains experience variable disease progression and severity [46], [47]. Disease progression in these models also differ depending upon the strains of parasite used [48]. In general, Balb/c mice are more susceptible to infection compared to C57Bl/6 mice in terms of increased parasitemia and mortality given a similar parasite challenge [49]. Kinetic analysis of cytokine production by lymphocytes provides evidence that resistance in C57Bl/6 mice is associated with increased early production of IFN-γ [49]. Further studies show that immunization protocols capable of inducing polarized Th1 but not Th2 responses are able to protect Balb/c mice against *T. cruzi* challenge, but transfer of CD4 T cell alone was not enough to confer protection to naïve mice [50]. While these studies indicate that Th1 versus Th2 responses are correlated with protection versus resistance, the complete profile of effector mechanisms leading to increased resistance in Th1 skewed mice has yet to be elucidated.

In the present study, we analyzed the humoral response to *T. cruzi* experimental infection of susceptible Balb/c versus resistant C57Bl/6 mice. We infected Balb/c and C57Bl/6 mice with isolates from Y-strain parasite that generated similar timing and magnitude of initial patent parasitemia. The kinetics, magnitude, and isotype of the parasite-specific and total circulating antibody responses during acute *T. cruzi* infection were examined. We further evaluated humoral responses in the spleen and the association of parasite-specific antibody secreting cells (ASC) with activation of B cells and expansion of B cell subsets, as well as the expansion and activation of splenic T cells. The combined results of this study demonstrate that resistant C57Bl/6 mice generated parasite-specific humoral responses that were associated with decreased hypergammaglobulinemia, differential kinetics of splenic B cell activation and B cell subset expansion, improved T cell help, and early IFN-γ production compared to more susceptible Balb/c mice.

## Materials and Methods

### Ethics Statement

All animals were handled in strict accordance with good animal practice as defined by the relevant national animal welfare bodies and all animal work was approved by the Institutional Animal Care and Use Committee of the University of Pittsburgh (Assurance Number A3187-01).

### Parasites and Mice

**Figure 1:**
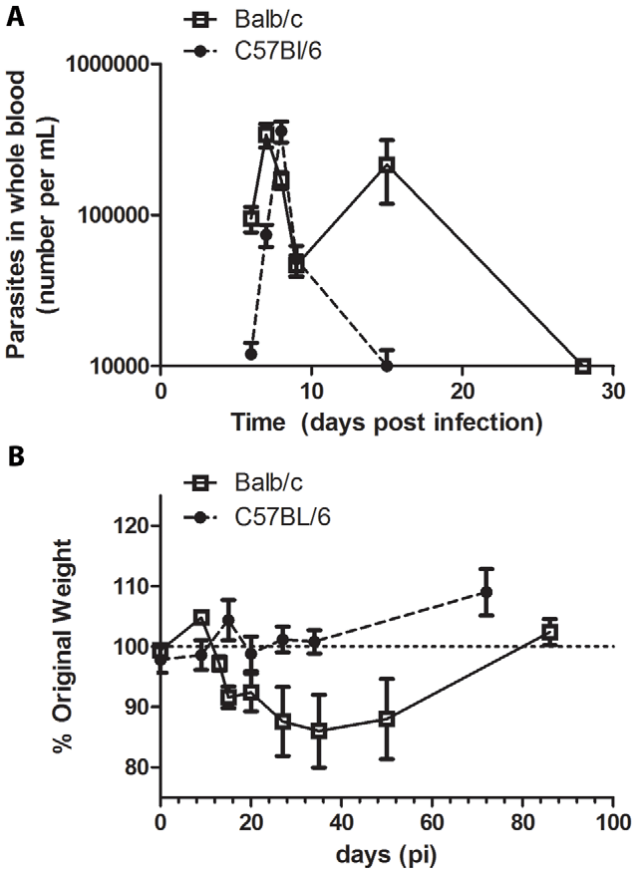
Disease severity and parasitemia in Balb/c and C57Bl/6 mice. Mice were injected with tissue culture trypomastigotes (0.25×LD50; i.p.). *A*, Parasitemia levels were analyzed in mouse blood at 6–9, 15, and 28 days post-infection (n≥5 mice per group). *B*, Mouse weights were collected at multiple time-points post-infection and the change from baseline determined for each mouse (n = 5 mice per group).

Balb/c and C57/Bl/6 mice were obtained from Jackson Laboratories and maintained in specific pathogen free housing. Mice husbandry and procedure protocols were reviewed and performed in accordance with the University of Pittsburgh IACUC. Y-strain parasites were grown in NIH 3T3 fibroblast cells and harvested by standard technique [51]. Briefly, 3T3 fibroblasts were infected with *trypomastigote* parasites and cultured for 2 days at 37°C (5% CO_2_) in DMEM (Gibco) supplemented with 10% FBS, 10 mM HEPES, 0.2 mM sodium pyruvate, and 50 µg/mL gentamicin, to allow infection of the growing cells. On day 3, the media was exchanged and the culture was moved to a 34°C incubator and maintained under anerobic conditions until harvest of the tissue derived trypomastigotes (TCT) from the culture supernatant. To establish models of infection for analysis of kinetics and magnitude of parasite-specific humoral immunity versus polyclonal B cell activation in resistant C57Bl/6 and susceptible Balb/c mice, we evaluated experimental infection using two variants of *T. cruzi* Y-strain (Y-US and Y-Br). The LD_50_ was established for each model, using intraperitoneal injection of TCT in PBS plus 1% glucose. Survival curves for these infections indicated that the Balb/c mice were much more susceptible to the Y-Br variant, with an approximately 1000 fold higher LD_50_, but succumbed to infection with different kinetics than did C57Bl/6 mice (Figure S1 *A*). At similar LD_50_, parasitemia was delayed in Y-Br-infected Balb/c compared to Y-Br-infected C57Bl/6 (Figure S1 *B*). In contrast, using the Y-US variant, Balb/c mice experienced initial peak parasitemia comparable with C57Bl/6 mice given a similar LD_50_ dose of the Y-Br variant (Fig 1*A*). In both mouse strains, a dose of parasite equal to approximately 0.25×LD_50_ was used to allow direct comparison of humoral responses in a sub-lethal infection of relatively resistant versus susceptible mice. The approximate 0.25×LD_50_ dose corresponded to 2.5×10^5^ Y-US in Balb/c mice and 6.25×10^3^ Y-Br in C57Bl/6 mice. Control mice were inoculated with the same dose of parasite after it had been heat-inactivated at 56°C for 25 minutes. Parasitemia was monitored by applying blood diluted 1/4 in RBC lysis buffer (150mM NH_4_Cl, 10mM NaHCO_3_, 115uM EDTA) to a hemocytometer and counting at 400×. Blood was collected at multiple time-points post infection for analysis by ELISA.

### rCRP Cloning

A full-length cDNA encoding the *T. cruzi CRP* was isolated by reverse transcription-PCR as previously described [52]. The *T. cruzi CRP* cDNA encoding the mature protein (starting at nucleotide 303) was subcloned into the pTrcHis expression vector (Invitrogen). *E. coli* strain SURE (Stratagene) were transformed with pTrcHis-CRP DNA for recombinant protein production with a histidine tag, as previously described [51]. For eukaryotic expression, CRP was cloned into pcDNA3 with the glycosylphosphatidylinositol (GPI) anchor signal sequence from human decay accelerating factor (daf), [53].

### Gene-Gun Immunization

pcDNA3_*CRP* DNA was purified from *E. coli* using endotoxin -free Mega prep kits (Qiagen). The DNA was coated on 1.0 µm gold particles (Bio-Rad) and loaded into Tefzel tubing (Bio-Rad) [54]. DNA (8 µg) was administered by Helios Gene-gun (Bio-Rad) at 400 psi, in two shots per mouse on shaved abdomen [55]. Four inoculations were performed at monthly intervals. Immunized mice were bled after boosting and the blood processed to obtain sera.

### Protein Purification

Expression of recombinant protein was induced by isopropyl-β-D-thiogalactoside (IPTG) (IBI Scientific, Peosta, IA) in transformed *E. coli* and the cells harvested by centrifugation (6,000 RCF, 10 min., 4°C). The resulting cleared lysate was prepared under denaturing conditions and bound to cobalt metal affinity resin according to the Talon instruction manual with slight modifications (Clontech, Mountain View, CA). During binding of lysate to resin, 5–10mM imidazole (Sigma) was added into the binding buffer (50mM Tris, 300mM NaCl, pH 7.2). Protein was bound to the resin in batch for 2 hours. The bound protein was further washed and packed into a disposable column. Imidazole (150mM) was used to elute bound protein. Protein concentration in eluted fractions was determined by Bradford assay.

### Quantitative IgM and IgG ELISA

4 HBX Immulon ELISA plates (Thermo Scientific) were coated with 100ng of goat-anti-mouse Ig antibody (SouthernBiotech) overnight at 4°C, washed, and blocked with 1% milk in T-PBS (0.05% Tween-20, 1×PBS), washed and stored at −20°C until use. Serum samples were stored at less than −20°C then thawed and maintained at 4°C during ELISA analysis. Serum was diluted in milk-T-PBS and applied to coated plates. A standard curve was generated with mouse IgM or IgG (SouthernBiotech). Goat anti-mouse IgM or IgG conjugated with HRP was used as the secondary antibody (SouthernBiotech). After incubation with secondary antibody, plates were washed and developed with OPTEIA (BD) and analyzed for color change (OD_450_). Standard curve fit and calculation of unknowns was performed using Prism software (GraphPad).

### rCRP and Whole Parasite Specific ELISA

For rCRP analysis, 4 HBX Immulon ELISA plates (Thermo Scientific) were coated with 100 ng of purified protein and incubated overnight at 4°C. For whole parasite ELISA, plates were coated with 2×10^5^ heat-inactivated TCT per well and incubated overnight at 4°C. Plates were washed and blocked and stored at −20°C until used. Mouse serum was diluted in block and applied to ELISA plates overnight at 4°C. Plates were washed and developed with the appropriate secondary antibody. The estimated reciprocal endpoint titer (RET) was determined graphically based on the OD_450_ values from equivalent dilution of pooled mouse pre-immunization serum samples. RET was defined as the first dilution with a value below the pre-immune OD_450_ plus SD (two to three replicates). Alternatively, equivalent units, determined by dividing the OD450 of test serum by the OD450 of pre-immune serum, were reported at a single dilution of sera.

### Antibody Secreting Cell (ASC) ELISPOT

Spleens were processed for single cells, by gentle mashing in a 40µM cell strainer, treated with RBC lysis buffer (150mM NH_4_Cl, 10mM NaHCO_3_, 115uM EDTA), washed with 1×PBS, and suspended in cRPMI. Multiscreen HTS 96-well ELISPOT plates (BD Biosciences) were coated with 2.5 µg/mL of rCRP or 5 µg/mL of goat anti-mouse Ig (SouthernBiotech) and incubated overnight. ELISPOT plates were washed with T-PBS and blocked with cRPMI for 2 hrs. Blocking media was removed and cells were plated into the ELISPOT plates (5 wells per sample) at several dilutions. After 5–6 hrs, the cells were washed off with PBS (3×) followed by T-PBS (3×). Secreted antibodies were detected by incubating with anti-mouse IgG conjugated to biotin (16 hrs, 4°C), washing with T-PBS (3–4×), incubation with avidin-peroxidase complex (30 min, RT)(Vector Laboratories, Burlingame, CA), washing with T-PBS (3×) and PBS (3×), followed by incubation with AEC ELISPOT substrate (8 min, RT)(BD Biosciences). The reaction was stopped by washing with PBS. Spots were analyzed using ImmunoSpot image acquisition 4.5 and ImmunoSpot 5.0 Professional DC software (ImmunoSpot). The frequency of ASC per 10^6^ splenocytes was determined [56], [57], [58].

### Flow Cytometry

Splenocytes were isolated, counted, and plated at 5×10^5^ cells per well in 96 well plates. Cells were collected by centrifugation (500×g, 5 min, 4°C) and washed with FACS staining buffer (1×PBS with 2.5% FCS, 1% goat serum, and 1% human AB serum). 10^7^ cells per mL were incubated with fluorescently labeled abs, diluted in FACS buffer, for 20 min on ice or 5 min at 4°C. Abs used for staining included anti-CD19 (MB19-1), CD3 (17A2), CD69 (H1.2F3), CD86 (GL1), CD21 (eBio4E3), CD23 (B3B4), CD4 (RM4-5, L3T4), CD8a (53-6.7), PanNK (DX5), CD95 (15A7), CD95L (MFL3). For determining population gating on cells with multiple stains, florescence minus one controls were used. For analysis of surface activation, B and T cells were counter stained and doublets were excluded to ensure only the reported lymphocyte population was analyzed. Antibodies were purchased from BD Biosciences or eBioscience. Data were collected on an LSRII (BD Biosciences) and analyzed using FlowJo software (Tree Star). The data were analyzed using bi-exponential transformation for complete data visualization.

### Statistical Analysis

2-way ANOVA was used for comparing the two mouse models over time. Bonferroni post-test analysis, Student's *t* test, or Mann-Whitney tests were used for comparison of individual doses or time-points, either between infected and control mice or between models.

## Results

### Balb/c and C57Bl/6 Models of *T. cruzi* Infection

Balb/c and C57BL/6 mice were infected with a sub-lethal parasite dose (0.25 LD_50_) of TCT. The magnitude and timing of the initial peak parasitemia was similar in both models, although Balb/c mice experienced a second wave of parasitemia at day 15 post-infection that was not evident in the C57Bl/6 mice (Fig 1*A*). Furthermore, Balb/c mice displayed significant weight loss by day 15 post-infection (p = 0.005, Student's *t*-test) that did not rebound until after acute infection (>30 days post-infection), whereas C57Bl/6 mice maintained their weight over the course of infection (p = 0.009, 2-way ANOVA) (Fig 1*B*). These data show that given the same relative dose with similar early parasitemia kinetics, Balb/c mice remained more susceptible to the adverse effects of *T. cruzi* infection than were C57Bl/6 mice.

### Resistant Mice Have Decreased Hypergammaglobulinemia and Improved Parasite Specific Antibody Response

**Figure 2 pntd-0000733-g002:**
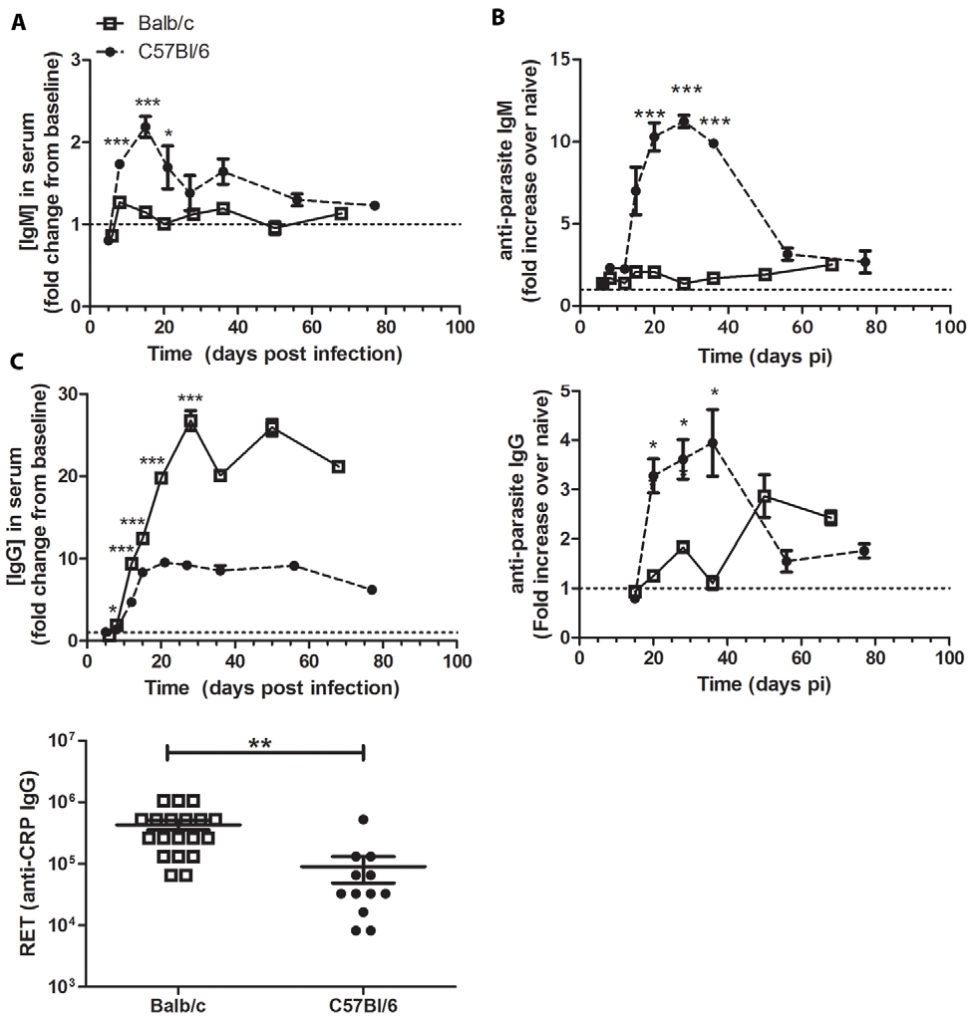
Circulating Total and CRP-specific IgM and IgG after *T. cruzi* infection. *A*, Infected mice (n = 5 per group) were bled at multiple time-points post-infection and their pooled sera analyzed for total IgM (top) and total IgG (bottom), with triplicate repeats for each time-point. *B*, Pooled sera were analyzed for CRP-specific IgM and CRP-specific IgG by ELISA in duplicate. *C*, Balb/c generate a higher titer CRP-specific IgG than do C57Bl/6 mice in response to genetic immunization (p = 0.002). Each data point represents one mouse, data pooled from several individual experiments. Balb/c are represented by open squares, solid line; C57Bl/6 mice by closed circles, dashed line. * p<0.05, *** p<0.001 by Student's *t* test comparing Balb/c and C57Bl/6 models.

To investigate systemic antibody responses during infection, serial pooled serum samples were collected and analyzed for levels of circulating IgG and IgM as well as antibodies specific to a *T. cruzi* surface antigen, *T. cruzi* complement regulatory protein (CRP), a member of the transialidase superfamily [51], [59], [60], [61]. The profiles of total IgM and IgG response in Balb/c and C57Bl/6 mice were significantly different during early infection (Fig 2*A*). C57Bl/6 mice had more circulating IgM after infection than did Balb/c mice. In contrast, Balb/c mice had significantly greater hypergammaglobulinemia post-infection than did C57Bl/6 mice (Fig 2*B*).

Parasite-specific antibody responses were evaluated by measurement of *T. cruzi* CRP-specific IgM and IgG following infection of Balb/c and C57Bl/6 mice. C57Bl/6 mice developed CRP-specific antibodies by day 20 post-infection (3.3±0.5 fold increase from baseline) (Fig 2*B*), whereas Balb/c mice had minimal CRP-specific responses until late in acute phase (Fig 2*B*). Delayed parasite-specific IgG response was also evident when Balb/c mice were inoculated with the low doses of the Y-BR variant (data not shown). In contrast, to the relative delay in CRP specific antibody responses in Balb/c versus C57Bl/6 mice during experimental infection, genetic immunization with CRP resulted in an improved CRP-specific IgG response in Balb/c mice compared to C57Bl/6 mice (Fig 2*C*). Taken together, these data suggest that the delayed CRP-specific antibody response in Balb/c mice compared to C57Bl/6 mice was not due to an inherent inability of Balb/c mice to respond to CRP antigen, but rather a host-pathogen interaction leading to diminished generation of parasite-specific antibody responses.

### A Th1 Skewed Early IFN-γ Response in Resistant Mice versus Delayed IFN-γ and Increased Th2 Cytokines in Susceptible Mice

**Figure 3 pntd-0000733-g003:**
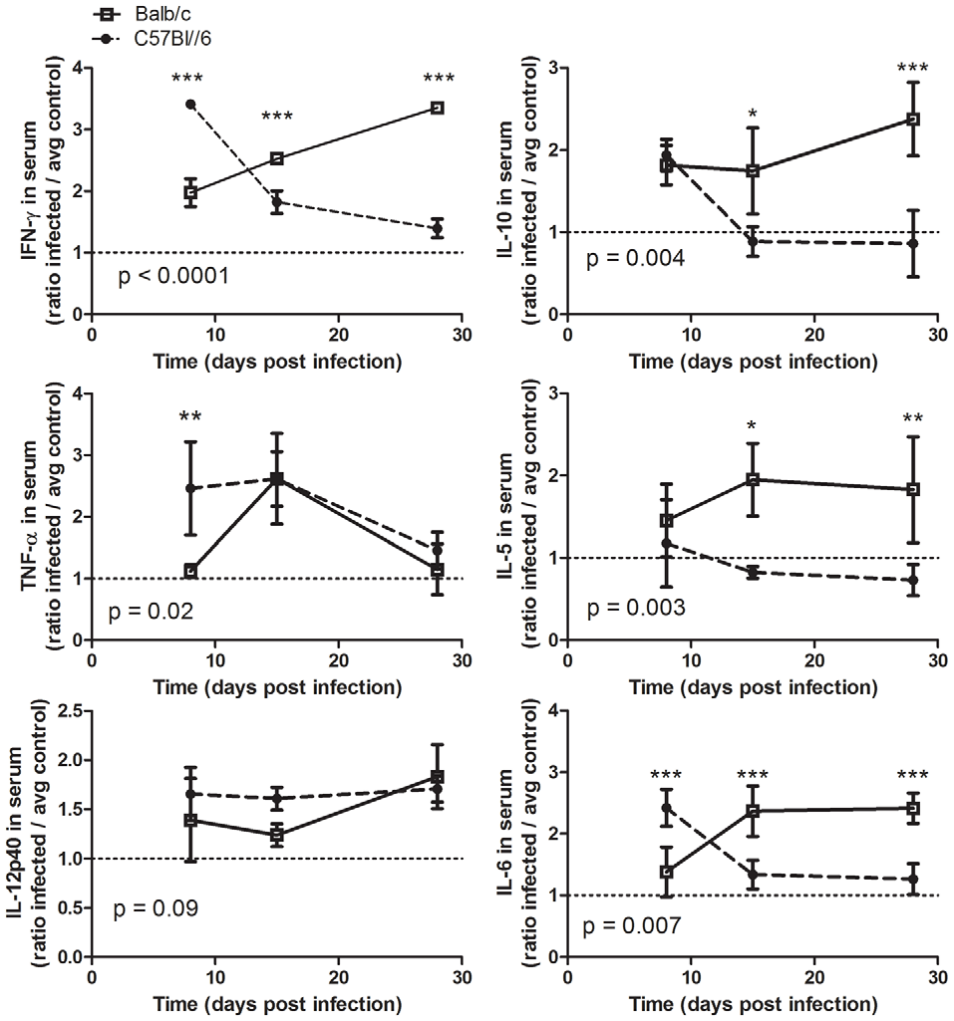
Cytokine profile after *T. cruzi* infection. Luminex analysis of cytokine levels in mouse serum samples at days 8, 15, and 28 post-infection. Values are reported as the concentration in infected mice relative to control mice receiving heat-inactivate parasite. Balb/c are represented by open squares, solid line; C57Bl/6 mice by closed circles, dashed line. * p<0.05, ** p<0.01, *** p<0.001 by Bonferroni post-test after 2-Way ANOVA comparing Balb/c and C57Bl/6 models (p values reported on graphs) and/or Student's *t* test.

Cytokines influence the generation of cellular and humoral responses to infection and can be differentially produced in resistant versus susceptible mice during *T. cruzi* infection [49], [50]. Cytokine production from T helper (Th) cells fall broadly into classification as T helper type 1 (Th1), T helper type 2 (Th2), or T helper 17 (Th17) categories: Th1 are defined by production of pro-inflammatory cytokines, particularly IFN-γ and TNF, Th2 by IL-4, IL-5, and IL-10, and Th17 cells by IL-17 [62]. To evaluate levels of circulating cytokines post-infection, multi-plex cytokine analysis was performed on serum samples from day 8, 15, and 28 post-inoculation, and compared to control mice inoculated with heat-inactivated parasites. The timing of IFN-γ, TNF-α IL-10, IL-5, and IL-6 responses were significantly different in Balb/c mice compared to C57Bl/6 (Fig 3). In C57Bl/6 mice, an early significant IFN-γ response was evident along with increased IL-6 and IL-10, which then decreased to near control levels by day 15 post-infection. In comparison to C57Bl/6 mice, Balb/c had delayed IFN-γ and IL-6 response, an early IL-10 that persisted to day 15 and increased by day 28 post-infection, and IL-5 levels that were significantly elevated by day 15 and remained so at day 28 post-infection. TNF-α levels increased earlier in C57Bl/6 mice than in Balb/c mice. In addition, Balb/c mice had IL-4 by day 28 post-infection, whereas Balb/c controls and C57Bl/6 mice (infected and control) did not produce detectable IL-4 in their serum (data not shown). These data indicate that C57Bl/6 responded to infection with an early Th1 biased cytokine response, whereas Balb/c mice responded to infection with a more Th2 skewed response.

### IgG1 and IgG2a Predominate in Total and Specific Response of Susceptible Mice versus a More Mixed Isotype Response in Resistant Mice

**Figure 4 pntd-0000733-g004:**
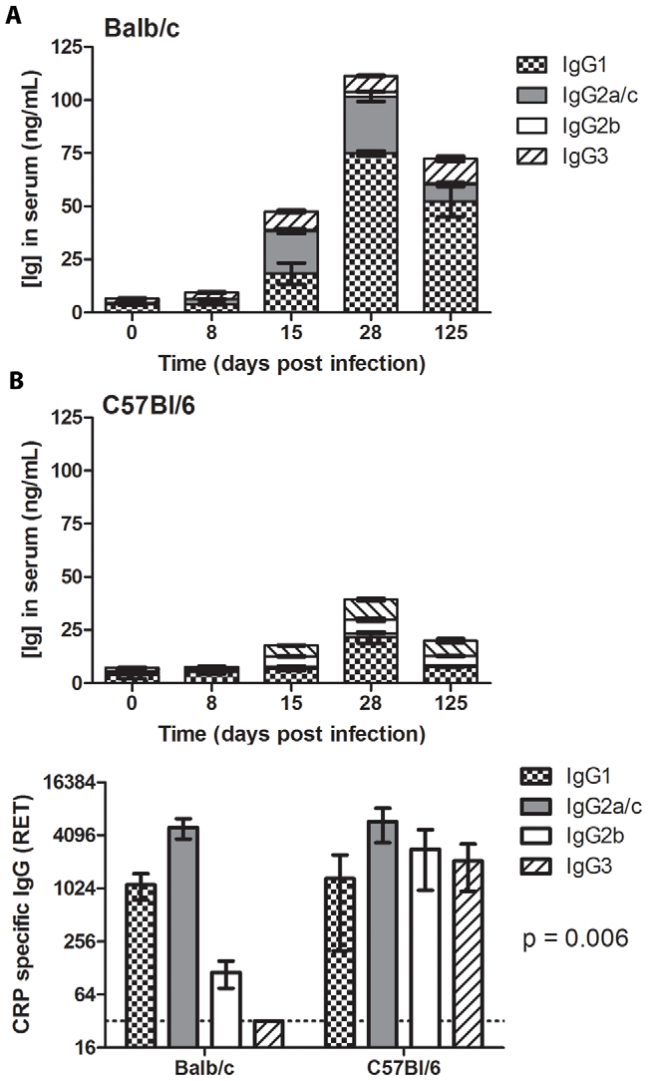
Antibody isotype and cytokine profile after *T. cruzi* infection. *A*, Serum samples from infected mice were analyzed by ELISA to determine the isotype of the total Ig at days 8, 15, 28, and 125 post-infection. For each isotype the profile was significantly different in Balb/c versus C57Bl/6 mice. IgG1 and IgG2a were increased in Balb/c mice compared to levels of IgG1 and IgG2a in C57Bl/6 mice, respectively (p<0.0001, 2-way ANOVA). IgG2b was increased in C57Bl/6 mice compared to Balb/c mice (p<0.0001, 2-way ANOVA). The profile of IgG3 showed an overall difference that was significant at days 15 and 125 post-infection (p<0.01 and p<0.001, respectively, by Bonferroni post-test; p = 0.0007 by 2-way ANOVA). Five mice were used per group per time-point. *B*, Serum samples from infected mice were analyzed for CRP-specific IgG isotype responses at day 28 post-infection by determining reciprocal endpoint titers (RET) using pre-infection sera. p = 0.006 by 2-way ANOVA comparing the CRP-specific isotype profile of Balb/c versus C57Bl/6 mice. Five mice were used per group.

Different IgG isotypes have been implicated in effective immunity and polyclonal B cell responses to *T. cruzi* [18], [19], [20], [21]. To compare the level of IgG isotype switching in Balb/c versus C57Bl/6 mice, serum samples were analyzed over the course of infection for concentration of IgG1, IgG2a (Balb/c) or IgG2c (C57Bl/6), IgG2b, and IgG3 [63]. The serum levels for each isotype were significantly different in Balb/c versus C57Bl/6 mice over the time-points tested (day 8 through 125, post-infection) (Fig 4*A*). IgG1 and IgG2a were increased in Balb/c mice compared to C57Bl/6 mice (p<0.0001 for each, 2-way ANOVA). IgG2b was increased in C57Bl/6 mice compared to Balb/c mice (p<0.0001, 2-way ANOVA). The profile of IgG3 showed an overall difference that was significant at days 15 and 125 post-infection (p<0.01 and p<0.001, respectively, by Bonferroni post-test; p = 0.0007 by 2-way ANOVA). Serum samples from infected mice were analyzed for CRP-specific IgG isotype responses at day 28 post-infection, revealing a significant difference in the isotype profile of parasite specific IgG (p = 0.0006, 2-way ANOVA), with predominantly IgG1 and IgG2a produced in Balb/c, with a mixed response including IgG2b and IgG3 in C57Bl/6 mice (Fig 4*B*).

### Resistant Mice Generated Parasite Specific IgG ASC; Susceptible Mice Generated Non-Specific IgG ASC and Have Increased Numbers of B Cell Blasts

**Figure 5:**
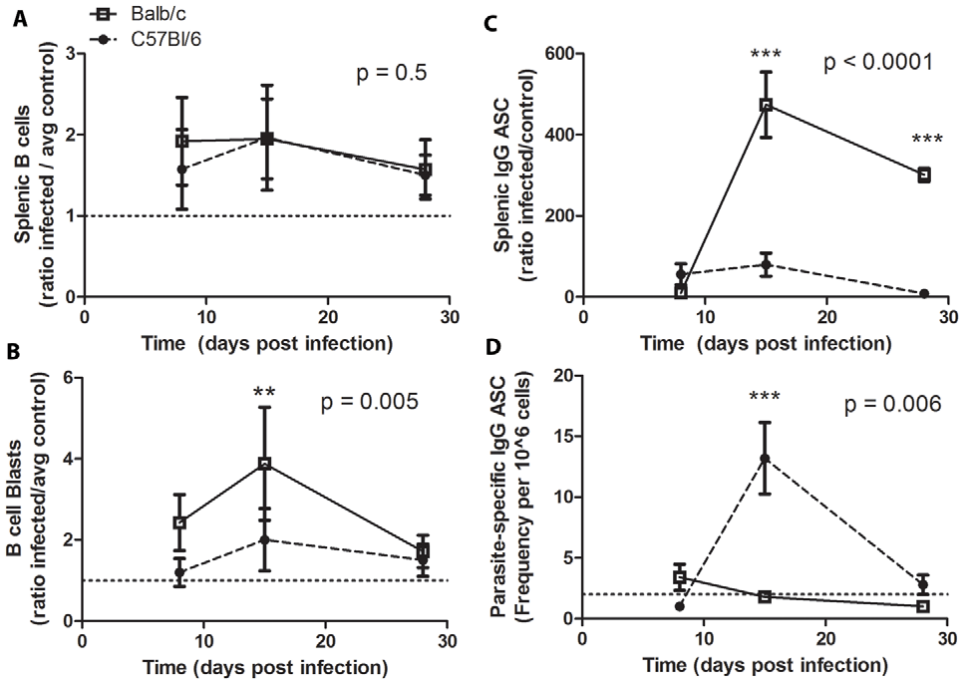
Splenic B cell expansion with or without parasite specific ASC. *A*, Splenocytes were stained and analyzed by flow cytometry for enumeration of B cells. *B*, B cells were analyzed by forward and side scatter for blast formation. These plots show the total splenic B cells in infected mice compared to the total number of splenic B cells in control mice of the same genotype. *C*, B cell ELISPOT analysis was completed to enumerate total IgG ASC from spleens of infected and control mice (n = 5 mice per group per time-point). The results are presented as the ratio of infected to average control of the same genotype. *D*, B cell ELISPOT analysis was used to enumerate the frequency of CRP-specific IgG ASC in the spleen of infected mice (n = 5 mice per group per time-point). Balb/c are represented by open squares, solid line; C57Bl/6 mice by closed circles, dashed line. ** p<0.01, *** p<0.001 by Bonferroni post-test after 2-Way ANOVA comparing Balb/c and C57Bl/6 models (p values reported on graphs).

Acute *T. cruzi* infection has been reported to induce expansion of B and T cell subsets in the spleen without generation of parasite specific responses [28], [64]. To determine whether resistant and susceptible mice display altered humoral responses in the spleen, splenocytes were analyzed for total B cells and large B cell blasts, as well as total and parasite-specific IgG antibody secreting cells (ASC) after *T. cruzi* infection (Fig 5). *T. cruzi* infection led to expansion of splenic B cells in both Balb/c and C57Bl/6 mice compared to controls by day 8 post infection (1.92±0.60 fold increase p<0.01 and 1.57±0.54 fold increase p = 0.04, respectively). (Fig 5A). B cell blasts, defined by their increased size and granularity, were increased to a greater extent in Balb/c mice compared to C57Bl/6 mice post-infection (Fig 5B). Balb/c mice had B cell blasts levels ≥2.4 fold higher than controls at day 8 and 15 post infection (p = 0.002 for both time-points), with levels falling to 1.7±0.9 by day 28 post-infection (p = 0.03). In contrast, C57Bl/6 mice first experienced a significant increase in total B cell blasts compared to controls (2.0±0.9, p = 0.02) at day 15 post-infection. Elevated levels of total IgG ASC were seen in both models but to a greater extent in Balb/c mice (Fig 5*C*). Furthermore, the increase in IgG ASC in Balb/c mice was not associated with an increase in parasite-specific ASC measured by CRP-specific ASC (Fig 5*D*). In contrast, C57Bl/6 show a parasite-specific response in the spleen at day 15 post-infection (Fig 5*D*). These data indicate that splenic B cells in Balb/c mice were more susceptible to *T. cruzi* induced polyclonal activation, but were less capable of generating a specific response to *T. cruzi* antigen. C57BL/6 mice were able to generate specific IgG ASC against a parasite specific protein, indicating an improved humoral response in the spleens of these mice. Furthermore, these data demonstrate that while there were similar changes in total B cell numbers in these two mouse models during *T. cruzi* infection, these changes were associated with different outcomes and that these differences in B cell effector function were also linked to phenotypic differences, such as blast formation.

### Analysis of B Cell Activation by Surface Expression of CD69, CD86 Fas, and FasL

**Figure 6 pntd-0000733-g006:**
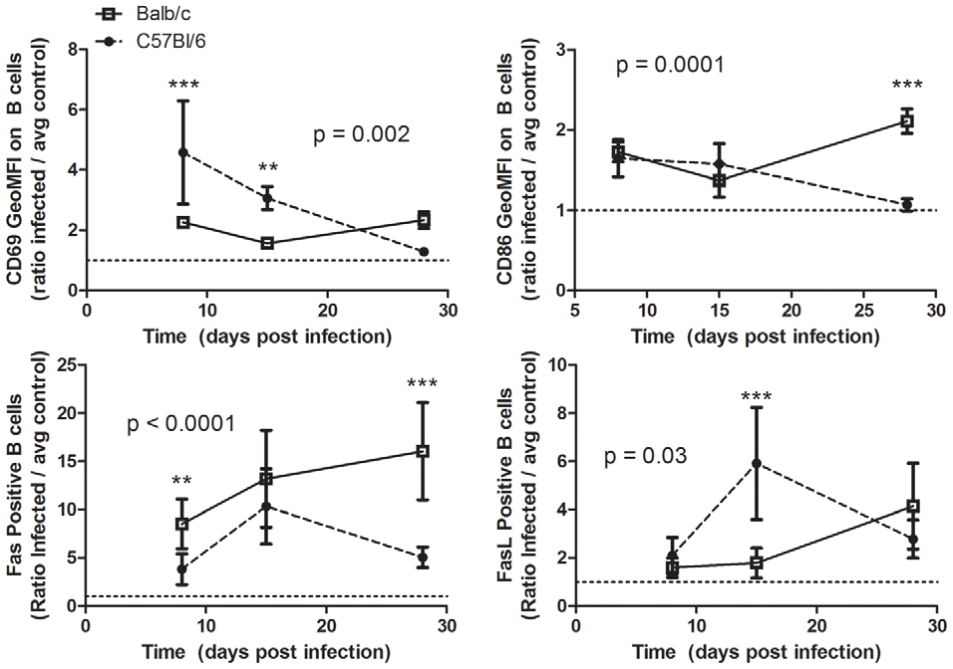
Expression of CD69, CD86, Fas, and FasL on splenic B cells. Splenocytes were harvested and stained for analysis by flow cytometry at multiple time-points post-infection with *T. cruzi* in Balb/c and C57BL/6 mice. Balb/c are represented by open squares, solid line; C57Bl/6 mice by closed circles, dashed line. ** p<0.01, *** p<0.001 by Bonferroni post-test after 2-Way ANOVA comparing Balb/c and C57Bl/6 models (p values reported on graphs) and/or Student's *t* test.

To analyze activation status of splenic B cells post-infection, flow cytometry was performed to measure surface expression of CD69, CD86, CD95 (Fas) and CD95L (FasL) at days 8, 15, and 28 post-inoculation, comparing expression on B cells in infected mice to control mice receiving heat-inactivated parasite (Fig 6). CD69 was increased post-infection in both models, but with different profiles (p = 0.0002, 2-way ANOVA). C57Bl/6 mice initially had higher CD69 on the surface of B cells (4.6±2.2 fold higher than controls at day 8 p = 0.002), which then decreased over the course of infection. Balb/c mice maintained approximately a 2 fold elevation in CD69 expression (2.3±0.2 fold higher than controls at day 8 p<0.0001). CD86 was increased post-infection in both models, but with different profiles (p = 0.0001, 2-way ANOVA). CD86 was initially elevated on B cells in both strains, then decreased in C57Bl/6 mice and increased in Balb/c mice at d28 post-infection. In both models, Fas and FasL positive B cells were increased post-infection, but with different profiles (p<0.0001 and P = 0.03, respectively, 2-way ANOVA). Fas and FasL on B cells in infected C57Bl/6 mice increased between d8 and d15, then declined between d15 and d28. In contrast, Fas and FasL on B cells in infected Balb/c mice increased between d15 and d28 post-infection. Together, these data indicate that B cells were differentially activated in susceptible versus resistant mice.

### Differential B Cell Subset Profiles in Resistant versus Susceptible Mice

**Figure 7:**
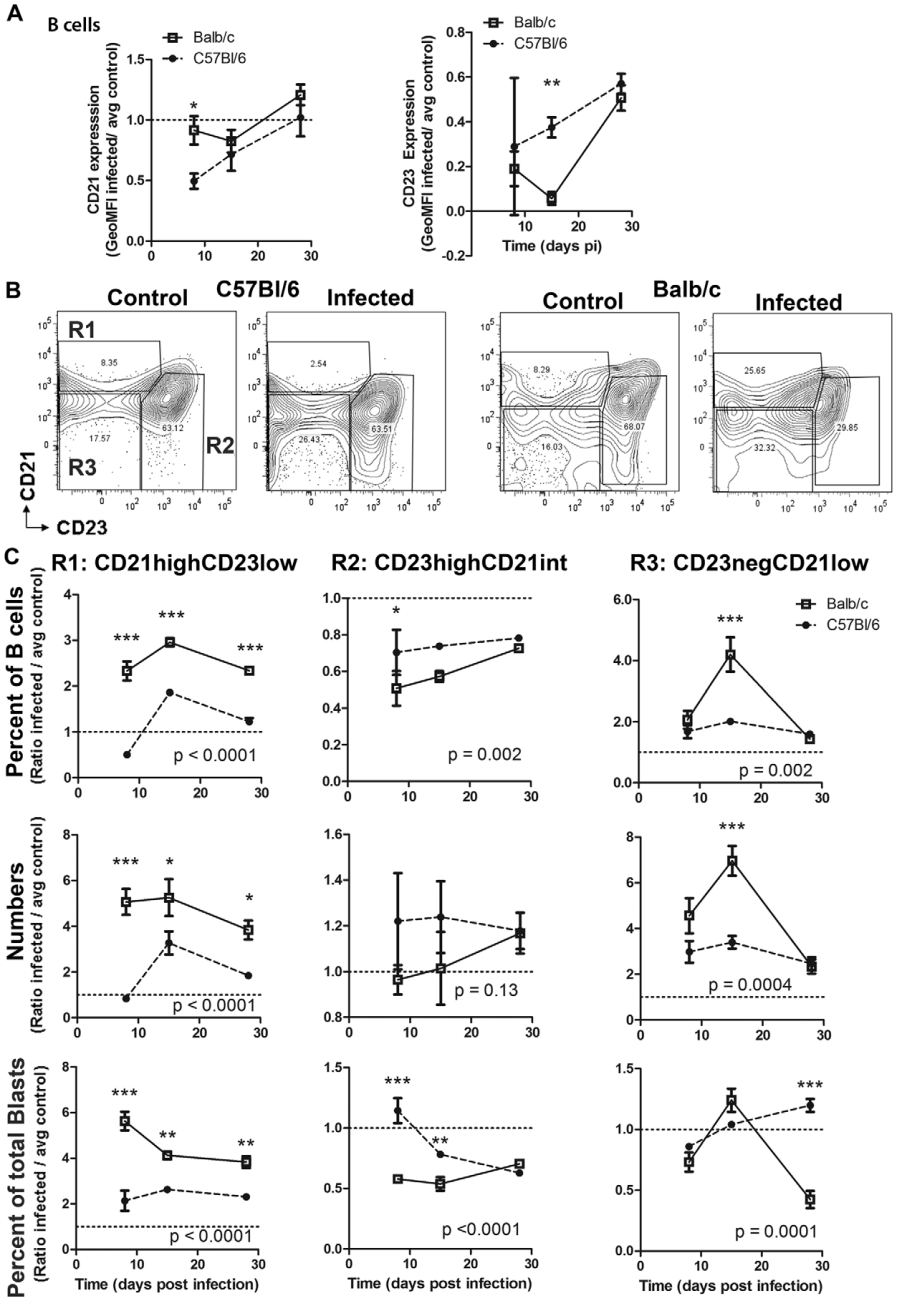
Splenic B cell subset changes. Splenoctyes were harvested from infected and control mice at days 8, 15, or 28 post infection and analyzed by surface markers for B cell populations. *A*, Analysis of CD21 and CD23 expression levels over the course of infection. *B*, Representative contour plots from day 8 post-infection show gates used for subset determination. *C*, These plots show the relative change in splenic B cell phenotype between infected and control mice. The first row graphs show percent of B cells accounted for by each of the subsets, the second row graphs show the absolute numbers for each population, the third row graphs shows the percent of B cell blasts accounted for by each of the subsets. In these sets of graphs, the y-axis shows the ratio of infected to average control (n = 5 mice per group per time-point). Graphs for a given population are underneath that title. Balb/c are represented by open squares, solid line; C57Bl/6 mice by closed circles, dashed line. Reported p values indicate the result of 2-way ANOVA analysis between Balb/c and C57Bl/6 mice. * p<0.05, ** p<0.01, *** p<0.001 by Bonferroni post-test after 2-Way ANOVA comparing Balb/c and C57Bl/6 models (p values reported on graphs) and/or Student's *t* test.

FO and MZ B cells are functionally and phenotypically distinct B cell populations within the spleen occupying distinct locations in the spleen. MZ B cells are poised at the marginal sinus of the spleen, and these cells are a source of natural antibody and T-independent (TI) responses [65], [66]. In contrast, FO B cells are located in spleen follicles and respond to antigen in a T cell dependent (TD) manner [39]. To analyze changes in B cell subsets as defined by phenotype, B cell gates were determined based on controls and naïve mice, using published population statistics as a guide [39]. B cell subset populations were defined as CD19^+^CD21^high^CD23^low/−^ (MZ B cells), CD19^+^CD21^int^CD23^+^ (FO B cells) and CD19^+^CD21^low^CD23^low/−^ cells (transitional/newly formed B cells, or cells that may have lost CD21 and CD23 expression due to activation). This gating strategy has been used to demonstrate changes in phenotypically defined MZ and FO B cell splenic subsets due to infection with other microbes [36], [37], [67], [68], [69]. While CD21 and CD23 levels have been shown to be modulated by virus [70], in particular in B cells infected with virus [71], the extent that other microbes modulate expression of these markers has not been determined. Analysis of CD21 and CD23 levels on B cells from *T. cruzi* infected mice indicates that the expression of both markers decreased overall on B cells by day 8 post-infection in resistant C57Bl/6 mice, after which expression increased over the course of infection; CD21 remained relatively unchanged in susceptible Balb/c mice at day 8 and 15, then slightly increased at day 28 post-infection, while CD23 levels were decreased at every time-point analyzed (Fig 7*A*). The changes in expression of these markers on total B cells were then reflected in apparent changes in these phenotypically defined B cell subsets post-infection with *T. cruzi* in both mouse models. Representative plots of B cell subsets for C57Bl/6 and Balb/c mice at d8 post-infection are shown in Figure 7*B*.

In Balb/c mice, the percentage of CD19^+^CD21^int^CD23^+^ (phenotypically defined as FO B cells) within the B cell gate decreased to 36±17 percent of controls (p<0.0001) and in C57Bl/6 decreased to 74±41 percent of controls (p = 0.02), which was a significant retention of FO B cells in C57Bl/6 mice compared to Balb/c mice (Fig 7*C*), although absolute numbers of FO B cells were not significantly different from controls in either model. Analysis of FO B cells within the total B cell blast population indicates that FO B cell blasts represent a higher proportion of the total B cell blasts at day 8 and 15 post-infection in resistant C57Bl/6 mice than in susceptible Balb/c mice (p<0.01 for both time-points) (Fig 7*C*).

CD19^+^CD21^high^CD23^low/−^ splenic B cells (defined as MZ by phenotype) responses also differed in Balb/c mice and C57Bl/6 mice. The percentage of B cells with the MZ phenotype was significantly different in Balb/c versus C57Bl/6 mice (p<0.0001, 2-way ANOVA). While in Balb/c mice, MZ B cells accounted for an increased percentage of the total B cells at d8, in C57Bl/6 mice, the percentage of MZ B cells were decreased (Fig 7*C*). The percentage of B cells accounted for by MZ remained elevated at all three time-points for Balb/c mice. C57Bl/6 experienced an increased percentage of MZ B cells at d15 post-infection, which then declined by d28 post-infection. Absolute numbers of MZ B cells were significantly different in Balb/c versus C57Bl/6 mice over the course of infection (p<0.0001) (Fig 7*C*). By d8 post-infection, MZ B cell numbers were increased 4.5±2.3 fold above controls in Balb/c mice (p = 0.01). In contrast, MZ B cell numbers were similar to controls in C57Bl/6 mice (0.83±0.29, p = 0.4). MZ B cell numbers were increased at d15 post-infection in C57Bl/6 and then declined, but remained elevated above controls at d28 post-infection. Analysis of CD19^+^CD21^high^CD23^low/−^IgM^high^ B cells showed these same trends of altered B cell numbers (data not shown). The percent of total B cell blasts that were represented by the MZ phenotype followed this same pattern of increased representation in the B cells from susceptible Balb/c mice, significantly more than in the resistant C57Bl/6 mice (p<0.0001).

CD19^+^CD21^low/−^CD23^low/−^ B cells numbers and percentages within the B cell gate were expanded in both models by day 8, Balb/c mice experienced further increased numbers and levels measured at d15 post-infection, whereas C57Bl/6 mice did not (p<0.0001, p = 0.002, numbers and percentage of B cells, respectively)(Fig 7*C*). These cells represented similar amounts of the total B cell blasts for both models until day 28 post-infection, when the numbers of blast cells represented by this subset decreased in Balb/c mice compared to C57Bl/6 (p<0.001).

### Splenic T Cells Expand and Were Activated in Resistant Mice

**Figure 8:**
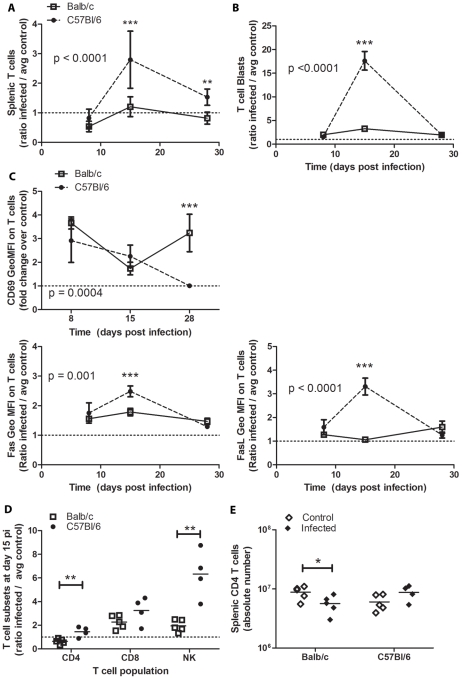
T cell expansion and activation in the spleen. Splenoctyes were harvested from Infected and control mice at days 8, 15, or 28 post-infection and analyzed by surface markers for T cell populations. Populations are reported as the ratio of absolute cell numbers or geometric mean florescent intensity (GeoMFI) on T cells in infected mice to the average absolute numbers for control mice. Data represent four-five infected mice per time-point. Balb/c are represented by open squares, solid line; C57Bl/6 mice by closed circles, dashed line. * p<0.05, ** p<0.01, *** p<0.001 by Bonferroni post-test after 2-Way ANOVA comparing Balb/c and C57Bl/6 models (p values reported on graphs) and/or Student's *t* test.

To determine the expansion and activation of splenic T cells, the number of total T cells and T cell blasts were assessed at days 8, 15, and 28 post-inoculation, comparing T cells in infected mice to control mice receiving heat-inactivated parasite (Fig 8*A,B*). C57Bl/6 mice had increased T cells at d15 that decreased but remained elevated by d28 (Fig 8*A*). This coincided with higher levels of T cell blasts at d15 (Fig 8*B*). Balb/c mice did not have increased T cells, although T cell blasts were increased at d15. CD69 expression on T cells was significantly increased compared to controls in both models and to a similar extend at day 8 post-infection (3.6±0.3 for Balb/c and 2.9±1.3 for C57Bl/6, p<0.01 compared to control, p = 0.12 between models). CD69 expression diverged at day 28 post-infection, when C57Bl/6 T cells had CD69 levels comparable to controls and Balb/c T cells had 3.2±0.8 fold increased CD69 expression compared to controls (p = 0.0002) and compared to the previous time point (1.9±2.6 p = 0.004). Fas and FasL profiles were significantly different on T cells from Balb/c versus C57Bl/6 mice (Fig 8*C*), especially at d15. CD4 and NKT cells levels were significantly higher in C57Bl/6 mice compared to Balb/c mice at d15 post-infection (Fig 8*D*). C57Bl/6 mice maintained or slightly increased splenic CD4 numbers at d15 pi, whereas Balb/c mice had decreased CD4 numbers post-infection (Fig 8*D*).

**Figure 9:**
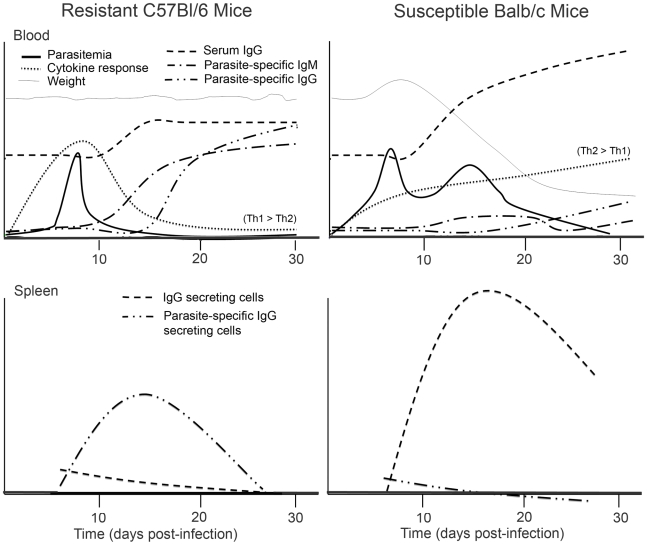
Differential antibody and cytokine responses associated with resistance to *T. cruzi*. These plots model responses in *T. cruzi* infected C57Bl/6 and Balb/c mice compared to control mice inoculated with heat-inactivated parasite (original data in Fig 1–5) and show the association of parasite-specific antibody responses in the blood and spleen with decreased polyclonal antibody responses and differential cytokine expression (kinetics and quality summarized here). Serum cytokine levels (see Fig 3 for details) in C57Bl/6 compared to controls were IFN-γ>TNF-α = IL-6>IL-10 with significantly elevated IL-12p40 and undetectable IL-5. The overall cytokine profile of Balb/c mice was IL-10≥IL-5, IL-6, and TNF-α, with increased IFN-γ and IL-12p40 later during infection (Fig. 3). The isotype of serum IgGs for C57Bl/6 mice were IgG1>IgG3>IgG2b>IgG2c, whereas Balb/c mice were IgG1>IgG2a>IgG3>IgG2b (Fig 4). Weight; the y-intercept is 100% and the origin is approximately 80% of the baseline. Parasitemia; the origin is the limit of detection (10^4^ parasites/mL) and peak parasitemia approximately 5×10^5^ parasites/mL. For cytokine responses, serum IgG, and parasite-specific IgM/G in both the blood and the spleen, the y-axis is the relative change in infected versus control mice and is to scale for each measure (i.e. directly compare IgG between C57Bl/6 and Balb/c mice). The plots for serum IgG are offset above the others for clarity.

The results presented in this study demonstrate that increased polyclonal B cell antibody responses were associated with decreased parasite-specific humoral immunity and increased disease susceptibility during *T. cruzi* infection (Figure 9). Hypergammaglobulinemia was more pronounced in susceptible Balb/c mice and was associated with delayed generation of parasite-specific antibodies. The parasite-specific humoral immunity in the resistant C57Bl/6 mice was concomitant with a Th1-skewed cytokine burst. In comparison, delayed and then maintained Th2-skewed cytokine production in Balb/c mice was associated with polyclonal B cell activation. B cell activation in resistant mice was associated with low levels of total ASC and appreciable parasite-specific ASC in the spleen. Total B cell expansion and activation in the spleen of susceptible mice was associated with the development of high levels of antibody secreting cells (ASC) without detection of parasite-specific response. Figure 9 provides a model for the association of polyclonal versus parasite-specific antibody with disease severity and cytokine responses in resistant and susceptible mice. Furthermore, analysis of B cell surface markers (CD69, CD86, Fas, FasL, CD21, CD23) indicated differential profiles in the context of polyclonal versus parasite-specific activation in resistant C57Bl/6 versus susceptible Balb/c mice. B cells with the marginal zone (MZ) phenotype were differentially expanded in the spleen of resistant C57Bl/6 mice in association with parasite-specific responses. MZ-like B cells were expanded early and remained elevated in susceptible Balb/c mice. Changes in T cell surface marker expression (Fas, FasL, CD69) and subsets (CD4, CD8, NK) were observed in resistant versus susceptible mice and associated with differential humoral responses.

## Discussion

As in many experimental models of infection, there are significant differences in susceptibility to pathogens among mouse strains; however the host-pathogen interactions that account for these differences are not completely clear. In the case of *T cruzi* experimental infection, protective cytotoxic CD8 responses and Th1 responses have been well characterized [72], [73], [74], [75]. The extent of B cell activation during *T. cruzi* infection and its association with effective parasite-specific immunity versus adverse polyclonal responses remains unclear.

It has been previously been shown that *T. cruzi* induces polyclonal B cell activation during acute infection and that B cell mitogens are expressed by infectious trypomastigotes [22], [28], [76], [77]. It has been postulated that this aberrant B cell response may contribute to a delayed parasite-specific humoral response, thus promoting infection [12], [14], [33], [76]. In this study, we examined the humoral responses of two murine strains with differing susceptibility to *T. cruzi* infection to 1) determine whether induction of polyclonal B cell activation was associated with increased disease susceptibility and 2) determine whether changes in circulating cytokines, splenic B cell function and phenotype, and splenic T cell activation and subset expansion were associated with polyclonal versus parasite-specific humoral responses.

Analysis of total circulating IgG and IgM indicated that resistant C57Bl/6 mice demonstrated increased total IgM followed by an IgM response to a parasite specific antigen (CRP) (Fig 2). The IgM responses in C57Bl/6 mice developed as parasites were cleared from circulation and were followed by a rise in total IgG and then parasite-specific IgG. In contrast, Balb/c mice had very little change in total IgM in the blood post-infection and minimal parasite-specific IgM responses. In contrast to the lack of IgM response, Balb/c mice had an increased hypergammaglobulinemia response compared to C57Bl/6 mice. The increased hypergammaglobulinemia evident in Balb/c mice was associated with a further delay and diminished parasite-specific IgG response compared to C57Bl/6. This lack of a robust IgG response to a parasite specific antigen (CRP) in Balb/c mice during experimental infection with *T. cruzi* was not due to an inherent inability to generate a response to this *T. cruzi* antigen, as evident by the magnitude of the IgG response to CRP after genetic immunization in Balb/c versus C57Bl/6 mice. Rather, these data suggest underlying host-parasite interactions that determine the balance of hypergammaglobulinemia versus the development of parasite specific responses.

As Th responses play a role in the generation of specific immune responses, as well as polyclonal B cell activation [21], we hypothesized that the differential humoral immune response to *T. cruzi* infection may be associated with differential production of cytokines in these two models. Analysis of circulating cytokines post-infection confirmed the skewing of Balb/c mice toward Th2 responses and the C57BL/6 mice toward Th1 responses (Fig 3). These data are in agreement with previous reports that early Th1 IFN-γ responses are associated with resistance to infection [50], [78] and that Th2 cytokines, especially IL-4 and IL-10 are associated with susceptibility to infection [34], [79], [80], [81], [82], [83], [84]. The results of the present study show that early Th1-skewed cytokine burst was coincident with control of parasitemia and preceded the generation of parasite-specific humoral immunity in resistant C57Bl/6 mice (Fig. 9). Susceptible Balb/c mice had a delayed cytokine response, which was Th-2 skewed including IL-5 and IL-4 production, and was associated with delayed parasite-specific responses and exacerbated hypergammaglobulinemia (Fig. 9). The apparent delay in cytokine responses and the lack of detectable IL-4 until day 28 post-infection in susceptible Balb/c mice may have been due to rapid consumption of these cytokines, especially IL-4, rather then lack of their production. Overall, these data suggest that the early Th-1 cytokine burst in susceptible mice may have dampened parasite-mediated polyclonal B cell activation, allowing for improved parasite-specific humoral responses, whereas sustained Th-2 cytokine production in susceptible mice may exacerbate parasite-induced polyclonal B cell activation. As B cells have been shown to produce cytokines in response to parasite-derived mitogenic factors [85], it is possible that B cell activation may account for some of the observed increases in cytokine levels post-infection with *T. cruzi*.

Cytokines can drive antibody production and influence isotype switch [86].The differential production of cytokines in susceptible Balb/c versus resistant C57Bl/6 mice was associated with differential total and parasite-specific IgG isotype response over the course of acute infection (Fig 2, 3, and 9). IgG1 and IgG2a made up the majority of the hypergammaglobunemia in Balb/c mice, whereas C57Bl/6 mice had a much lower IgG1 and IgG2a responses and increased total IgG2b (Fig 4). While IgG1 was the most elevated isotype in both mouse models, IgG2a showed a much greater increase from baseline in Balb/c and IgG2b in C57Bl/6 mice. C57Bl/6 mice experienced isotype switching of parasite-specific antibodies to IgG1, IgG2c, IgGb, and IgG3. In contrast, the Balb/c mice had limited isotype switching; the parasite-specific response remained predominately IgG1 and IgG2a out to day 28 post-infection. These data confirm the previously reported trend towards differential IgG isotype responses in resistant versus susceptible mice [19], [20] and further support the association of parasite-specific IgG2, particularly IgG2b, with increased resistance to *T. cruzi* [10], [87], [88].

Previous studies have reported B cell expansion in the spleen during *T. cruzi* infection [12], [20],[21],[28],[31],[89]. This expansion is increased when intact CD4 T-cell responses are present [21] and evidence shows that it is dissociated from parasite specific responses [12], although some studies report both total and specific B cell responses. In the present study, we performed a detailed analysis of the B cell response to infection and found that although total numbers of B cells were expanded to similar extents in susceptible and resistant mice, the outcome of these expansions were different in terms of production of total and specific IgG ASC, activation status by CD69, CD86, Fas/FasL expression, and B cell subset expansion (Fig 5–7).

Splenic B cell expansion in susceptible Balb/c mice was associated with increased numbers of B cell blasts, as well as with increased numbers of IgG ASC, without appreciable expansion of parasite specific IgG ASC. In contrast, B cell expansion in resistant C57Bl/6 mice led to moderately increased B cell blast formation, moderate to low levels of IgG ASC, and the formation of parasite-specific IgG ASC (Fig 5). CD69, a marker of lymphocyte activation [88], was moderately increased on Balb/c splenocytes throughout infection, whereas in C57Bl/6 mice, CD69 was differentially expressed over the course of acute infection, with an early peak expression that was two fold higher on C57Bl/6 B cells compared to Balb/c B cells. The early rise in CD69 on C57Bl/6 B cells preceded parasite-specific ASC formation, after which CD69 levels decreased (Fig. 6). These data demonstrated that transient high level CD69 expression preceded parasite-specific B cell activation, while moderate sustained CD69 expression was associated with polyclonal B cell activation. Previous studies have indicated a role for increased CD86 on B cells during *T. cruzi* infection in leading to increased immunoglobulin production through interaction with NK cells [90]. In this study, CD86 expression on B cell was not significantly different between these two models early in infection, but diverged later with increased levels of Balb/c B cells at day 28 post-infection concomitant with peak hypergammaglobulinemia in these mice.

Recent studies have indicated that *T. cruzi* causes parasite specific B cells to undergo Fas-FasL mediated fratricide [30]. Fas is also expressed on B cells during germinal center reactions, without leading to apoptosis (reviewed in [91]). Our data shows that Fas and FasL were differentially expressed on the surface of B cells in C57Bl/6 versus Balb/c mice during *T. cruzi* infection (Fig 6). In resistant C57Bl/6 mice, increased Fas and FasL positive B cell numbers were associated with CRP-specific IgG ASC. Fas and FasL were also increased on T cells in conjunction with this CRP-specific IgG response in the spleen. Together, these data suggest that the Fas/FasL expression on B cells in C57Bl/6 mice was associated with a productive, germinal-center like, reaction in the spleen. In contrast, elevated Fas/FasL positive B cell numbers in susceptible Balb/c mice were not associated with a parasite-specific response. Rather, the sustained elevated expression of these death ligands may have limited the non-specific expansion of B cells in these mice through B-cell fratricide, as increased activation and blast formation did not lead to overwhelming expansion of B cells during acute infection.

To better understand which B cells account for the observed increase in splenic B cells during *T. cruzi* infection, we evaluated B cell subsets based on CD21 and CD23 expression of CD19+ B cells (Fig 7). These markers have previously been used to define splenic B cell subsets ex vivo during microbial infection [36], [37], [67], [68], [69]. As the expression of these markers may be modulated by activation induced by infection, the definition of these subsets is not definitive. Future studies are necessary to define the affect of *T. cruzi* infection on these B cell subsets in vivo. For the purposes of this study, B cell subset populations were defined as CD19^+^CD21^high^CD23^low/−^ (MZ B cells), CD19^+^CD21^int^CD23^+^ (FO B cells) and CD19^+^CD21^low^CD23^low/−^ cells. In Balb/c mice, B cells retained CD21 expression, but decreased CD23 expression, the low affinity IgE receptor. Susceptible mice had an apparent increase in B cells with the MZ phenotype within total B cells and B cell blasts that was associated with polyclonal rather than parasite-specific humoral responses (Fig. 5). Two possible explanations for the expansion of B cells with the MZ phenotype are that MZ B cells proliferated or parasite activation induced B cells differentiation toward a MZ B cell phenotype. MZ B cells were not expanded early in infection in C57Bl/6 mice. Rather, transient expansion in MZ phenotype within B cells and B cells blasts coincided with the development of parasite-specific IgG ASC in resistant C57Bl/6 mice. The percentages of B cells with a FO phenotype decreased in both models, but were retained to a greater extend in the C57Bl/6 mice, although the variability between mice was quite high, leading to a lack of significant difference in absolute numbers of FO in C57Bl/6 versus Balb/c mice. The relative amount of B cells with the FO phenotype within the B cell blast population was higher during early infection of C57Bl/6 mice compared to Balb/c mice, suggesting that in the context of parasite-specific response B cell blasts retain the FO phenotype to a greater extend than in the context of polyclonal B cell activation. Analysis of CD19^+^CD21^low^CD23^low/−^ cells indicated that Balb/c mice experienced increased expansion of these cells compared to C57Bl/6 mice. The expansion of transitional B cells without parasite-specific ASC in susceptible mice suggests that this expansion results from polyclonal B cell activation rather than parasite-specific humoral immunity. The relatively low representation of this population in the total number of B cell blasts in susceptible mice suggests that perhaps decreased CD23 expression, rather than proliferation led to the relative expansion of this subset compared to controls. All together, these data indicate differential changes in B cell subset phenotype in resistant versus susceptible mice. Further studies are needed to fully define the functional consequence of these apparent B cell subset changes and their contribution to humoral response during *T. cruzi* infection.

To evaluate whether these differences in humoral response were associated with differences in T cell dynamics in resistant versus susceptible mice, bulk splenic T cells were analyzed for expansion, blast formation, and expression of Fas and FasL (Fig 8). T cell expansion was coincident with the generation of parasite specific IgG responses at day fifteen post-infection in resistant mice, but remained near control levels in during the polyclonal B cell activation in susceptible mice at this same time-point. CD69 expression on total T cells was similar early during infection for both models. Later in infection, T cells from infected Balb/c mice experienced a second wave of CD69 activation, which was coincident with clearance of parasite from circulation and the first detection of parasite-specific IgG in circulation, although these levels did increase much until later on during infection (after day 36). Low Fas and FasL expression in Balb/c mice suggests that the contribution of T cells to control of B cell numbers via apoptosis was minimal in these mice. The expression of Fas and FasL on T cells coincided with parasite-specific B cell responses in C57Bl/6 mice, suggesting they may have formed a productive association. Further analysis of T cells at this time-point indicated that resistant mice had significantly higher levels of CD4 and NK T cells than susceptible mice, both of which can provide B cell help [92]. While previous studies show that lack of CD4 T cells led to decreased polyclonal B cell activation [21], these results suggest that maintenance of CD4 and increased NK T cells may also be important for directing the efficacy of the specific humoral response to parasite antigen. Activation of NK T cells has previously been linked to increased resistance to *T. cruzi*, but depends upon the presence of CD8 and CD4 T cells, as well as IFN-γ production [93], [94]. The increased numbers of NK T cells in resistant C57Bl/6 mice is particularly intriguing as they may provide B cell help through direct interaction (CD40L) or rapid cytokine responses, especially IFN-γ, which was produced to a much greater extent in these resistant mice [95]. These data provide rationale for further studies to fully define the contribution of CD4 and NK T cells to polyclonal versus parasite-specific humoral immunity during *T. cruzi* infection of susceptible versus resistant mice.

While susceptibility and resistance of Balb/c and C57Bl/6 mice have been documented and explored in terms of cellular and cytokine responses to *T. cruzi* experimental infection [2], [49], [78], [79], [96], [97], [98], [99], [100], [101], humoral responses in these studies have been largely neglected. This is the first study to examine both polyclonal and specific humoral responses in these mice in the context of equivalent initial parasitemia. Taken together, the results in this study support the hypothesis that polyclonal B cell activation leading to hyper-IgG responses are associated with increased disease susceptibility and highlights the importance of host-parasite interactions in development of humoral responses to *T. cruzi*. By further characterizing associations between Th1 and Th2 responses, the development of polyclonal versus parasite-specific humoral responses, and the potential contribution of B cell subsets to these processes, this present study provides a more detailed understanding of the development of effective versus detrimental humoral responses during *T. cruzi* infection. Furthermore, these results have implications for vaccine design in *T. cruzi*, as host genetic biases that lead to differential polyclonal B cell activation may have profound effects on the development of humoral immunity to *T. cruzi* target antigens [14], [102], [103].

## Supporting Information

Figure S1 Survival curves and parasitemia profile of mice infected with Y strain variants. Mice (5–10 per dose) were injected i.p. with TCT derived parasites and monitored for survival and for parasite numbers in tail blood. A, Top: Babl/c mice inoculated with the indicated doses of Y-Br variant. Middle: C57Bl/6 mice inoculated the indicated doses of Y-Br variant. Bottom: Balb/c mice inoculated with the indicated doses of Y-US variant. B, Parasitemia profiles for Balb/c mice inoculated with 10 (∼LD50) or 50 (2–3×LD50) Y-Br variant parasites, or C57Bl/6 mice with 10,000 Y-Br parasites (∼0.5LD50).
(0.24 MB DOC)
