# Supplementary material for: Specific Humoral Immunity versus Polyclonal B Cell Activation in Trypanosoma cruzi Infection of Susceptible and Resistant Mice
Source: PLoS Negl Trop Dis. 2010 Jul 6;4(7):e733. doi: 10.1371/journal.pntd.0000733 (PMC2897841; doi:10.1371/journal.pntd.0000733)
Supplement: Figure S1 — Survival curves and parasitemia profile of mice infected with Y strain variants. Mice (5–10 per dose) were injected i.p. with TCT derived parasites and monitored for survival and for parasite numbers in tail blood. A, Top: Babl/c mice inoculated with the indicated doses of Y-Br variant. Middle: C57Bl/6 mice inoculated the indicated doses of Y-Br variant. Bottom: Balb/c mice inoculated with the indicated doses of Y-US variant. B, Parasitemia profiles for Balb/c mice inoculated with 10 (∼LD50) or 50 (2–3×LD50) Y-Br variant parasites, or C57Bl/6 mice with 10,000 Y-Br parasites (∼0.5LD50). (0.24 MB DOC) [file pntd.0000733.s001.doc]

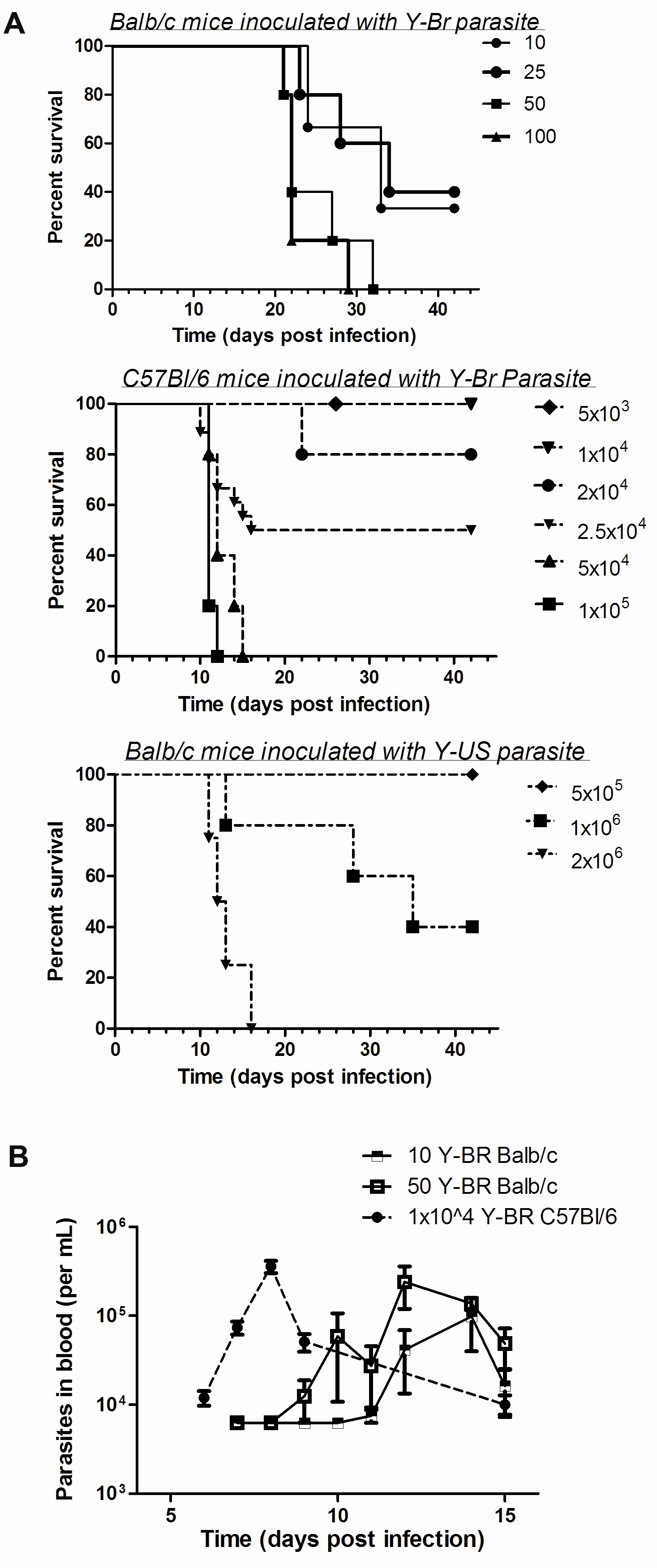


**Supplementary Figure 1: Survival curves and parasitemia profile of mice infected with Y strain variants.** Mice (5-10 per dose) were injected i.p. with TCT derived parasites and monitored for survival and for parasite numbers in tail blood. *A,* Top: Babl/c mice inoculated with the indicated doses of Y-Br variant. Middle: C57Bl/6 mice inoculated the indicated doses of Y-Br variant. Bottom: Balb/c mice inoculated with the indicated doses of Y-US variant. *B,* Parasitemia profiles for Balb/c mice inoculated with 10 (~LD50) or 50 (2-3xLD50) Y-Br variant parasites, or C57Bl/6 mice with 10,000 Y-Br parasites (~0.5LD50).
